# Trilobatin Protects Against Oxidative Injury in Neuronal PC12 Cells Through Regulating Mitochondrial ROS Homeostasis Mediated by AMPK/Nrf2/Sirt3 Signaling Pathway

**DOI:** 10.3389/fnmol.2018.00267

**Published:** 2018-07-30

**Authors:** Jianmei Gao, Shuang Liu, Fan Xu, Yuangui Liu, Chun Lv, Yan Deng, Jingshan Shi, Qihai Gong

**Affiliations:** ^1^Department of Clinical Pharmacotherapeutics, School of Pharmacy, Zunyi Medical University, Zunyi, China; ^2^Department of Pharmacology, Key Laboratory of Basic Pharmacology and Joint International Research Laboratory of Ethnomedicine, Ministry of Education, Zunyi Medical University, Zunyi, China

**Keywords:** trilobatin, hydrogen peroxide, PC12 cells, AMPK, Nrf2, Sirt3

## Abstract

Oxidative stress-induced neuronal cell damage is a crucial factor in the pathogenesis of mitochondria-associated neurological diseases. Therefore, elimination of overproduction of mitochondrial reactive oxygen species (mtROS) may be a potential strategy for prevention and treatment of neurological diseases. In the present study, the neuroprotective effects of trilobatin (TLB), a novel small molecule monomer derived from *Lithocarpus polystachyus* Rehd, and its underlying mechanisms were investigated *in vitro* using hydrogen peroxide (H_2_O_2_)-induced oxidative stress model in a neuron-like PC12 cell. The findings revealed that pre-treatment with TLB dramatically concentration-dependently suppressed H_2_O_2_-induced PC12 cells damage by enhancing cell viability, repressed reduction of mitochondrial membrane potential (MMP) and decreased mtROS overgeneration, thereby deferring cell apoptosis. Further study demonstrated that TLB not only increased the enzymatic activities of glutathione peroxidase (GPx), isocitrate dehydrogenase 2 (IDH2),superoxide dismutase 2 (SOD2) and deacetylation of SOD2, but also activated silent mating-type information regulation 2 homolog 3 (Sirt3) within the mitochondria and thereby upregulating forkheadboxO3a (FoxO3a), which regulated mitochondrial DNA genes, then led to improving complex I activity and adenosine triphosphate (ATP) synthesis. What’s more, TLB up-regulated p-adenosine monophosphate-activated protein kinase (AMPK) level, the expression of peroxisome proliferator-activated receptor-gamma coactivator 1 alpha (PGC-1α), and ERRα. Intriguingly, TLB failed to mitigate H_2_O_2_-induced PC12 injury in the presence of the AMPK inhibitor (Compound C), indicating that the beneficial effects of TLB on the regulation of mtROS homeostasis were reliance on AMPK -Sirt3 signaling pathway. Moreover, TLB also facilitated nuclear factor erythroid 2-related factor 2 (Nrf2) and promoted antioxidant gene expression in turn, and knockdown of Nrf2 by siRNA dramatically reduced the neuroprotective effects of TLB. Notably, AMPK inhibitor abolished the activation of Nrf2 and Sirt3, whereas, knockdown of Nrf2 blocked the upregulation of Sirt3, but it did not affect p-AMPK level. In conclusion, our findings demonstrate that TLB protects against oxidative injury in neuronal PC12 cells through regulating mtROS homeostasis in the first time, which is, at least partly, mediated through the AMPK/Nrf2/Sirt3 signaling pathway.

## Introduction

There is general recognition that considerable neurological diseases are characterized by a progressive death of neurons, accompanied with a decrease in antioxidant ability and/or an augment in oxidative stress, which is termed as a disequilibrium between the antioxidant enzymes system and the production of reactive oxygen species (ROS; Li et al., 2017). ROS, by definition, is generally taken to encompass the initial species generated by oxygen reduction including superoxide (${\text{O}}_{2}^{•-}$) or hydrogen peroxide (H_2_O_2_) as well as their secondary reactive products (Forman et al., 2015), and accumulating evidence supports a dual role of ROS as a “double-edged sword” that may be either beneficial or deleterious in living systems (Poprac et al., 2017). ROS at a low level appears to exert the beneficial effect in normal physiological conditions and multiple cellular signaling pathways. Nonetheless, it is widespread presumed that excessive ROS is a major contributor to the pathogenetic mechanisms underlying neurological diseases, such as Alzheimer’s disease, cerebral stroke, Parkinson’s disease (Kalogeris et al., 2014; Liu et al., 2015). Therefore, application of antioxidant which is termed as “a substance that, when present at a low concentration compared with that of an oxidizable substrate, inhibits oxidation of the substrate” to deter oxidative stress-induced neuron demise may be a potential strategy to fight neurological diseases (Halliwell and Gutteridge, 1995). Nevertheless, the exploitation of natural or synthetic antioxidant was considered promising therapeutic candidates for neurological diseases has been challenged in spite of promising preclinical data and a great deal of public interests.

Mitochondria are the sites of the tricarboxylic acid cycle and oxidative phosphorylation (OXPHOS), through which numerous adenosine triphosphate (ATP) are generated by the electron transport chain (ETC). Likewise, ROS are produced as byproducts of ETC, and incomplete decrease of molecular oxygen in the ETC leads to mitochondrial ROS (mtROS) generation (Shadel and Horvath, 2015). Hence, mitochondria are not only major sites of ROS production, but also tend to oxidative injury (Hung et al., 2018). Therefore, it is unsurprising that mtROS plays a critical role, and targeting to mtROS may be an important approach to affect cellular redox signaling pathways in the neurological diseases. A growing body of evidence indicates that silent mating-type information regulation 2 homolog 3 (Sirt3), localized to the mitochondrial matrix, is a mitochondrial nicotinamide adenine dinucleotide (NAD)^+^-dependent protein (Brown et al., 2013). Importantly, Sirt3 can not only orchestrate mitochondrial oxidative metabolism, but also modulate mtROS homeostasis by regulating the mitochondrial complexes such as complex I and complex III in the ETC, which are considered as the principal sites of mtROS production, as well as the main antioxidant enzymes of mitochondria including manganese superoxide dismutase 2 (SOD2), glutathione peroxidase (GPx) and isocitrate dehydrogenase 2 (IDH2; Liu J. et al., 2017). In addition, recent studies report that Sirt3 is activated by its upstream target of peroxisome proliferator-activated receptor gamma coactivator-1α (PGC-1α), which is directly regulated by adenosine monophosphate-activated protein kinase (AMPK), sequentially regulates mitochondrial antioxidant enzymes and forkheadboxO3a (FoxO3a)-regulated mitochondrial DNA transcription to limit the production of mtROS (Liu S.-G. et al., 2017). Notably, nuclear factor erythroid 2-related factor 2 (Nrf2), a principal key transcription factor, is recognized as a main stress responder that transcriptionally activates antioxidant and cytoprotective genes through binding to antioxidant response element (ARE) motifs in mtROS homeostasis. Intriguingly, Nrf2 is not only a regulator of Sirt3 expression, but also is activated by AMPK (Satterstrom et al., 2015; Lv et al., 2017). Thus, it reasonable to assume that AMPK/Nrf2/Sirt3 signaling pathway plays an imperative role in regulating the mtROS homeostasis.

**Figure 1:**
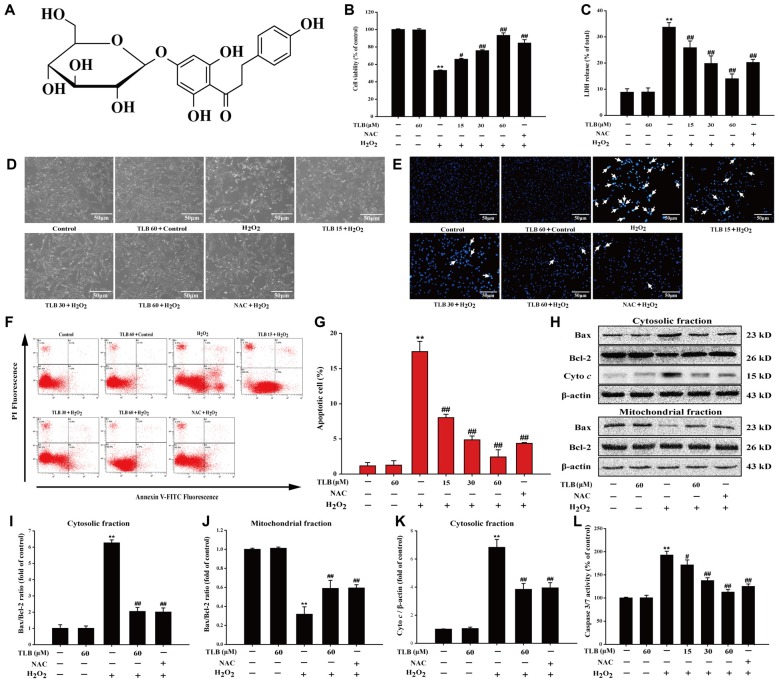
Trilobatin (TLB) protected PC12 cells against hydrogen peroxide (H_2_O_2_)-induced oxidative injury and cell demise. **(A)** The chemical structure of TLB. After pre-treatment with different concentrations of TLB, cells were treated with 400 μM H_2_O_2_ for another 48 h. **(B)** Cell viability was determined using 3-(4,5-dimethythiazol-2-yl)-2,5-diphenyltetrazolium bromide (MTT) assay. **(C)** Lactate dehydrogenase (LDH) release was determined using an LDH release assay. **(D)** The protective effect of TLB on H_2_O_2_-induced morphological changes in PC12 cells. Magnification, 200×; scale bar = 50 μm. **(E)** PC12 cells were stained by Hoechst 33342 dying, and apoptotic cells were labeled with white arrows indicating a nuclear shrinkage or intensive fluorescence. Magnification, 200×; scale bar = 50 μm. **(F)** Early apoptotic cells were detected by flow cytometry of annexin/propidium iodide (PI) double stained. **(G)** Bar graphs showed the quantification of early apoptotic cells. **(H)** Representative Western blot were shown for the expressions of Bax and Bcl-2 both in the cytoplasmic and mitochondrial fraction. **(I)** Quantitation of the ratio of Bax/Bcl-2 in the cytoplasmic fraction. **(J)** Quantitation of the ratio of Bax/Bcl-2 in the mitochondrial fraction. **(K)** Representative Western blot were shown for the expressions of Cyto *c*. **(L)** Quantification of caspase 3/7 activity was detected using caspase 3/7 activity assay. Data were presented as mean ± standard deviation (SD) of three independent experiments. **P < 0.01 vs. untreated control cells; ^#^*P* < 0.05, ^##^*P* < 0.01 vs. H_2_O_2_-treated cells.

Currently, natural antioxidants have gained extensive focus and exhibit beneficial effects on neurological diseases. Chinese herbs are plentiful resources to develop novel natural antioxidants. *Lithocarpus polystachyus* Rehd, a traditional Chinese folk medicine, has been used for a long time to prevent or treat various diseases (Hou et al., 2011; Zhou et al., 2013). Trilobatin (TLB; Figure 1A), a small molecule monomer, is considered as a novel bioactive compound of *Lithocarpus polystachyus* Rehd. Previous studies have demonstrated that TLB exhibited anti-oxidant and anti-inflammatory activities (Fan et al., 2015; Wang J. et al., 2016), but until now, the protective effect of TLB on oxidative stress-induced cellular mitochondrial dysfunction and apoptosis in neuron still remains a mystery. Under this scenario, the aim of this study was designed to assess whether TLB exerts salutary effects on H_2_O_2_-induced PC12 cell injury and its underlying mechanisms. In the current study, we found, for the first time, that TLB protected against H_2_O_2_-induced PC12 cell injury was through reducing mtROS production and apoptosis, which, at least in part, was via activation of AMPK/Nrf2/Sirt3 signaling pathway. These findings not only offer novel evidence concerning the neuroprotective effects of TLB, but also provide a new possible candidate for treating neurological diseases.

## Materials and Methods

### Materials

Analytical grade TLB was purchased from Guangdong Kedi Medical Technology Corporation (purity ≥98%), *N*-acetyl-Lcysteine (NAC, #A7250) was purchased from Sigma-Aldrich (St. Louis, MO, USA). Both agents were dissolved in dimethyl sulfoxide (DMSO) at 10 mM as stock solution and diluted in culture medium, respectively. The final concentration of DMSO in the media was not more than 1‰. H_2_O_2_ (#H1009), 3-(4,5-dimethythiazol-2-yl)-2,5-diphenyltetrazolium bromide (MTT, #M2128), rhodamine 123 (#R8004), 2’,7’-dichlorodihydrofluorescein diacetate (DCFH-DA, #D6883), isocitrate dehydrogenase (IDH) activity assay Kit (#MAK062) were purchased from Sigma-Aldrich (St. Louis, MO, USA). Caspase-Glo^®^ 3/7 Assay kit (#G8091) was obtained from Promega (Madison, WI, USA). The lactate dehydrogenase (LDH) Cytotoxicity Assay Kit (#A020-2), SOD2, (#A001-3), GPx, (#A005) assay kits were obtained from Jiancheng Bioengineering Institute (Nanjing, China). Hoechst 33342 (#C0031) were obtained from Solarbio Science & Technology (Beijing, China). Annexin V-FITC and propidium iodide (PI) apoptosis detection kit (#556570) was purchased from BD Biosciences (Mississauga, ON, Canada). MitoSOX Red (#M36008) and Mito Tracker green probe (#M7514) were obtained from Invitrogen (Eugene, OR, USA), Compound C (#ab120843), Mitochondria isolation kit for cultured cells (#ab110170), NAD/NADH quantification kit (#ab65348), Complex I enzyme activity microplate assay kit (Colorimetric; #ab109721), ATP synthase enzyme activity microplate assay (#ab109714), BCA protein quantification kit (#ab102536), anti-GP_X_-1 (#ab22604), anti-Sirt 3 (#ab189860), anti-IDH2 (#ab129180), anti-forkhead boxO3a (FoxO3a, #ab23683), anti-SOD2 (acetyl K68; #ab13533), anti-SOD2 (#ab13533), anti-uncoupling protein 2 (UCP2, #ab203244), anti-AMPK (#ab80039), anti-p-AMPK (#ab133448), anti-PGC-1α (#ab54481), anti-estrogen-related receptor-α (#ab76228), anti-Nrf2 antibody (#ab137550), anti-kelchlike ECH-associated protein 1 (Keap-1, #ab139729), anti-HO-1 (#ab68477), anti-NQO1 (#ab34173) antibodies were purchased from Abcam (Cambridge, UK). Nrf2 siRNA (r; # sc-156128) was purchased from Santa Cruz Biotech (Santa Cruz, CA, USA). Lipofectamine™ RNAiMAX transfection reagent (#13778075) and scrambled siRNA (#1841282) were obtained from Invitrogen (Eugene, OR, USA).

### Cell Culture

The neuron-like highly differentiated rat pheochromocytoma line PC12, which is a clonal cell line derived from a rat adrenal medulla, and has been broadly used as a cellular model of neuronal development and neurological diseases due to its neuronal properties, such as neurotransmitter release, neurite growth, response to nerve growth factor (Zhang et al., 2017). PC12 cells were obtained from American Type Culture Collection (Rockville, MD, USA). Cells were maintained in DMEM medium supplemented with 10% fetal bovine serum, penicillin (100 U/ml) and streptomycin (100 μg/ml), and incubated in a humidified atmosphere of 5% CO_2_ at 37°C.

### MTT Assay

Cell viability was determined using MTT assay as described in prior study. Briefly, 400 μM H_2_O_2_ treated PC12 cells (at a density of 2 × 10^4^ cells/well in 96-well plates) for 48 h was employed as oxidative stress model *in vitro* as described in our previous report (Gao et al., 2017). The PC12 cells were pre-treated with indicated concentrations of TLB for 1 h prior to expose to 400 μM H_2_O_2_ for another 48 h. Thereafter, the cells were incubated with MTT (5 mg/ml) for an additional 4 h. The medium was replaced with DMSO (150 μl) was added, and absorbance at 490 nm was evaluated by a microplate reader. Cell viability was expressed as the percentage of MTT reduction relative to the absorbance of the control cells. In parallel experiments, NAC, a thiol antioxidant, was used as a positive control for antioxidant capacity that suppresses cell demise, for compare with the effect exerted by TLB. The AMPK inhibitor 10 μM Compound C was added to the cell cultures 1 h or knockdown of Nrf2 prior to TLB treatment. Cell viability was expressed as a percentage compared with the control.

### LDH Assay

Release of cytosolic LDH into the extracellular medium was considered as an indicator of cytotoxicity, thus cytotoxicity was measured by an LDH assay kit according to the product manual. Briefly, PC12 cells were treated as mentioned above and then collected supernatant of each well and centrifuged at 2000× *g* for 20 min. Absorbance was read at 490 nm wavelength and all values of % LDH released were normalized to the control. In addition, cellular morphologic changes were observed using phase contrast microscopy.

### Hoechst 33342 Staining

In brief, PC12 cells were treated as mentioned above, these cells were washed with PBS, fixed with 4% formaldehyde and then stained with 10 μg/ml Hoechst 33342 for 20 min at room temperature in the dark. After washing with PBS, the nuclei were visualized using fluorescence microscopy (Olympus IX73; Olympus). The mean fluorescence intensity was quantized by the Image Pro Plus software.

### Annexin V-FITC/PI Staining

The cells were treated as described above, then washed with PBS and resuspended in the binding buffer containing Annexin V-FITC and PI for 15 min in the dark. Thereafter, the cells were detected by Navios flow cytometry (Beckman Coulter, Brea, CA, USA). The percentage of apoptotic cells was counted using Navios software (Beckman Coulter, Brea, CA, USA).

### Caspase 3/7 Activity Assay

PC12 cells were treated as previously mentioned, the caspase 3/7 activity was determined using the Caspase-Glo^®^ 3/7 Assay according to the manufacturer’s instructions (Zeng et al., 2017). PC12 cells were lysed in lysis buffer and centrifuged at 12,500× *g* for 5 min, thereafter 50 ml of 2× substrate working solution added into 15 ml of cell lysate were co-cultured in 96-well plates at room temperature for 30 min. Then the fluorescence intensity was detected using multimode reader with excitation at 490 nm and emission at 520 nm. All values of caspase 3/7 activities were presented as a percentage compared with the control.

### Measurement of ROS Level and Mitochondrial ${\text{O}}_{2}^{•-}$

PC12 cells were treated as mentioned above. Intracellular ROS generation was determined using DCFH-DA (Wedgwood et al., 2013). In brief, the cells were incubated with 20 μM DCFH-DA in DMEM for 30 min in the dark, rinsed twice with PBS solution and the fluorescence was observed and recorded using a fluorescent microscope at an excitation wavelength of 485 nm and an emission wavelength of 530 nm. Quantification of ROS level was evaluated using the Image Pro Plus software. All values of ROS level were expressed as a percentage compared with the control. Furthermore, the generation of mitochondrial ${\text{O}}_{2}^{•-}$ was determined using MitoSOX Red, a selective fluorescent indicator for detecting ${\text{O}}_{2}^{•-}$ generation within mitochondria. Briefly, PC12 cells were treated as mentioned above, cells were washed with balanced salt solution (HBSS) and stained with MitoSOX Red (5 μM) for 20 min in the dark at 37°C. Thereafter, the cells loaded with Mito Tracker green dye (200 nM) for 20 min under darkness at 37°C. Whereafter, the cells were washed with PBS and visualized by fluorescence microscopy (Olympus IX73; Olympus, Tokyo, Japan) with excitation/emission (510/580 nm) filters.

### Measurement of Mitochondrial Membrane Potential (MMP)

Rhodamine 123 dye was applied to monitor mitochondrial integrity as previously mentioned (Gao et al., 2017). After pre-treated with or without TLB, PC12 cells were stained with 2 μg/ml rhodamine 123 for 20 min in the dark, and then were washed with PBS. The density of green fluorescence (excitation 485, emission 595 nm) was visualized using fluorescence microscopy (Olympus IX73; Olympus), and then the mean fluorescence intensity was measured using the Image Pro Plus software. All values of green fluorescence density were expressed as a percentage compared with the control.

### Determination of Cellular NADH and NAD^+^ Concentrations

PC12 cells were treated as mentioned above. NAD^+^ and NADH concentrations were measured using the NAD/NADH Assay Kit according to the manufacturer’s instructions.

### Measurement of Mitochondrial Enzyme Activities

PC12 cells were treated as mentioned above. Briefly, mitochondria were isolated using a mitochondria isolation kit for cultured cells according to the manufacturer’s instructions. The sample protein concentrations were determined using BCA protein quantification kit and adjusted the final sample protein concentration is 5.5 mg/ml protein. Then, centrifuged the sample for 20 min at 12,000× *g* in a cold centrifuge. The mitochondrial enzyme activities of complex I, SOD2, GPx, IDH2 and the content of MDA were measured according to manufacture’s instruction of the assay kits, respectively.

### ATP Quantification

In brief, PC12 cells were treated and mitochondria were isolated as described above. The homogenized samples were centrifuged at 16,000× *g* for 20 min, the supernatant was used to detect ATP levels by luminescent ATP detection assay kit according to the manufacturer’s protocols. The activity of the ATP synthase enzyme was measured in absorbance at 340 nm wavelength. The activity rate is expressed as the change in absorbance at 340 nm/min/amount of sample loaded into the well.

### Extraction of Nuclear and Cytosolic Protein

Nuclear and cytosolic proteins were performed following a process reported previously with a nuclear extraction Kit (Jin et al., 2014). Nuclear and cytosolic proteins were fractionated according to the instructions of the manufacturer. Briefly, following treatment, cells were harvested and centrifuged at 300× *g* for 5 min at 4°C. The cell pellets were mixed with hypotonic buffer containing phosphatase and protease inhibitors. After 10 min incubation on ice, 10% Nonidet P-40 Assay Reagent was added to the cells. Nuclei were recovered by centrifugation (14,000× *g*, 30 s), and the supernatant was kept as cytoplasmic extract at −80°C until use. The nuclei were extracted with Nuclear Extraction Buffer for 30 min on ice. Insoluble material was removed by centrifugation at 14,000× *g* for 10 min. Finally, the supernatant was used as a nuclear extract (Yoo et al., 2017).

### Quantitative Real-Time PCR Analysis

Total RNA was extracted with the Trizol Reagent, which was reverse transcribed to cDNA with the PrimeScript™ RT Reagent Kit. Real-time PCR was performed on the CFX96 real-time PCR detection system (Bio-Rad Laboratories Ltd., Hertfordshire, UK) with specific primers and their sequences were listed as follows: GAPDH, 5’-AACGACCCCTTCATTGACCT-3’ and 5’-CCCCATTTGATGTTAGCGGG-3’; ATP6, 5’-TCATCAGAACGCCTAATCAG-3’ and 5’-GGGGTAAATGTATGGGGAAG-3’; CO1, 5’-ATTACAGCCGTCCTACTACT-3’ and 5’-TGGCCGAAGAATCAGAATAG-3’; ND2, 5’-AACCCTAACTTAACCCTCCT-3’ and 5’-TAGGGTTGAGCAGTTGTTTT-3’; ND5, 5’-ACAATCTAGTCCCTCTCACA-3’ and 5’-TGCTGTGAATAATGTGGTGA-3’; Cyto b, 5’-TGACCTTCCCGCCCCATCCA-3’ and 5’-AGCCGTAGTTTACGTCTCGGCA-3’; Sirt3, 5’-TGCACGGTCTGTCGAAGGTC-3’ and 5’-TGTCAGGTTTCACAACGCCAG-3’. In the reaction, 1 μl cDNA of each sample was mixed with SYBR^®^ PCR Master Mix according to the protocol of manufacture. And the PCR conditions: 95°C for 5 min, 40 cycles of 95°C for 10 s, 60°C for 45 s. The results were normalized to levels of GAPDH mRNA and expressed as the fold change (2^−ΔΔCt^).

### Western Blot

Western blot was performed as described previously (Gao et al., 2017). Briefly, the protein concentration was measured with BCA Protein Assay Kit. Samples with equal amounts of proteins were separated on 10% polyacrylamide gels, then the separated proteins were blotted onto PVDF membrane, and probed with selective antibody, respectively. The appropriate primary antibody: p-AMPK (1:1000), AMPK (1:1000), PGC-1 (1:1000), ERRα (1:1000), Sirt3 (1:1000), IDH2 (1:1000), FoxO3a (1:1000), ac-SOD2 (1:1000), SOD2 (1:1000), GPx-1 (1:1000), UCP2 (1:1000), Nrf2 (1:1000), Keap-1 (1:1000), HO-1 (1:1000), NQO-1 (1:1000). The intensity of the optical bands was quantified by ImageJ software.

### siRNA Transfection

Nrf2-siRNA was used to inhibit endogenous Nrf2 expression and a scrambled siRNA was used as a control. PC12 cells were seeded at 60% confluency into 6-well plates. Six microliter of Nrf2-siRNA and 18 μL lipofectamine™ RNAiMAX transfection reagent were diluted into 150 μL Opti-MEM I, and then gently mixed and incubated at room temperature for 15 min, then lipofectamine mixture was added to the Nrf2-siRNA mixture at a final concentration of 60 pM siRNA. Transfection of PC12 cells with scrambled siRNA served as a negative control. After incubation for 24 h at 37°C, 2 ml of complete medium with 10% FBS was added to the transfected cells to replace transfection solution and the cells were exposed to H_2_O_2_ at the same time. The knockdown of endogenous Nrf2 by siRNA was confirmed by Western blot. The transfected cells were cultured for 48 h, and then harvested for further analysis.

### Statistical Analysis

Data are expressed as mean ± standard deviation (SD). All experiments were performed in triplicate. The one-way ANOVA of variance followed by least significant difference *post hoc* test was applied to determine individual differences. Differences with *P* < 0.05 were considered statistically significant.

## Results

### TLB Concentration-Dependently Protected Against H_2_O_2_-Induced Injury in PC12 Cells

Previous study confirmed that the treatment of 400 μM H_2_O_2_ for 48 h resulted in significantly PC12 cell death (nearly 50%), thus, such concentration and incubation time were used for the following experiments. The results indicated that pre-treatment with 15, 30 and 60 μM of TLB markedly reduced H_2_O_2_-induced cell death in a concentration-dependent manner (66%, 76% and 93%, respectively; *F*_(6,14)_ = 65.682, *P* < 0.001; Figure 1B). Moreover, in parallel, pre-treated PC12 cells with 15, 30 and 60 μM of TLB significantly attenuated H_2_O_2_-induced LDH leakage (from 34% to 26%, 20%, 14%, respectively; *F*_(6,14)_ = 25.742, *P* < 0.001; Figure 1C). Furthermore, the protective effect of TLB was confirmed by morphologic observations, which showed that H_2_O_2_-treated cells resulted in dominant cellular shrinkage and floatation than that of control, whereas pre-treatment with TLB or NAC, the cells became normal morphology (Figure 1D). Moreover, nuclei condensation was observed in PC12 cells after exposure to 400 μM H_2_O_2_ by Hoechst 33342 staining, and pre-treatment with 15, 30 and 60 μM of TLB reduced these changes in nuclei (Figure 1E). In addition, apoptosis cell was detected in PC12 cells after exposure to H_2_O_2_ by annexin V/PI double staining and flow cytometry analysis. After H_2_O_2_ treatment, the rate of early apoptosis increased to 17.5%. However, pre-treatment with 15, 30 and 60 μM of TLB decreased the rate of early apoptosis to 8%, 5%, 2.5%, respectively (*F*_(6,14)_ = 160.148, *P* < 0.001; Figures 1F,G). It’s worth noting that, Bax expression was increased dramatically in the cytosolic fraction, whereas, decreased notably in the mitochondrial fraction in PC12 cells after treated with H_2_O_2_. However, H_2_O_2_ significantly decreased Bcl-2 expression in the cytosolic fraction, but there was slightly change of Bcl-2 expression in the mitochondrial fractions. Subsequently, the Bax/Bcl-2 ratio was significantly decreased in the mitochondrial fraction but increased in the cytosolic fraction in PC12 cells as a result of H_2_O_2_ treatment. However, pre-treatment with TLB suppressed the increase in H_2_O_2_-induced Bax/Bcl-2 ratio both in cytosolic and mitochondrial fractions (*F*_(4,10)_ = 79.661, *P* < 0.001; *F*_(4,10)_ = 94.141, *P* < 0.001). Additionally, TLB also decreased the cyto *c* in the cytosolic fraction (*F*_(4,10)_ = 47.137, *P* < 0.001; Figures 1H–K). Furthermore, caspase 3/7 activation is a main biomarker in the apoptosis progress of neuronal cells (Wang D. et al., 2016). The results revealed that treatment with H_2_O_2_ for 48 h increased caspase 3/7 activity to 193% than that of control. In contrast, pre-treatment with 15, 30 and 60 μM of TLB markedly decreased caspase 3/7 activation to 171%, 137%, 113%, respectively (*F*_(6,14)_ = 72.733, *P* < 0.001; Figure 1L). What’s more, TLB (60 μM) alone did not exhibit cytotoxicity and lead to apoptosis of PC12 cells, and the protective effect of TLB was same or higher than those exerted by an equivalent concentration of positive agent, a classical antioxidant, NAC. These results indicated that TLB protected against oxidative stress-induced apoptosis in H_2_O_2_-stimulated PC12 cells.

### TLB Restored H_2_O_2_-Induced Change of MMP

**Figure 2:**
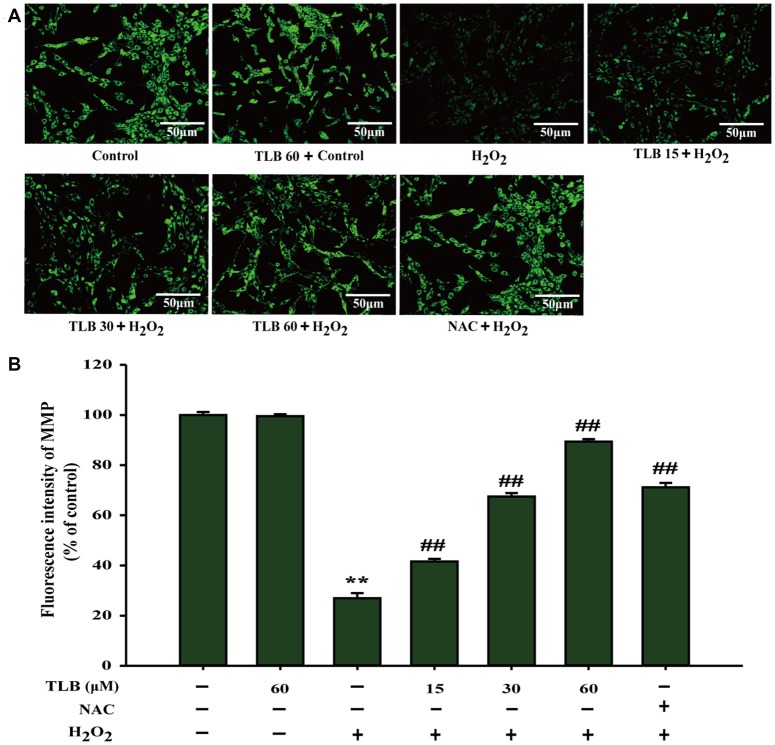
TLB mitigated H_2_O_2_-induced collapse of mitochondrial membrane potential (MMP) in PC12 cells. After pre-treatment with or without TLB for 1 h, cells were treated with 400 μM H_2_O_2_ for another 48 h. **(A)** MMP was determined by the rhodamine 123. Magnification, 200×; scale bar = 50 μm. **(B)** The mean fluorescence intensity of MMP was quantified using Image Pro Plus software. Data were presented as mean ± standard deviation (SD) of three independent experiments. **P < 0.01 vs. untreated control cells; ^##^*P* < 0.01 vs. H_2_O_2_-treated cells.

The mitochondrial membrane potential (MMP) was assessed by analyzing the green fluorescence intensity using rhodamine 123 staining as previous study (Gao et al., 2017). The results showed that pre-treatment with 15, 30 and 60 μM of TLB significantly inhibited the decrease of MMP induced by H_2_O_2_ (from 27% to 42%, 68%, 89%, respectively; *F*_(6,14)_ = 1312.749, *P* < 0.001; Figures 2A,B). These findings further confirmed that TLB protected against H_2_O_2_-induced PC12 cell death, at least partly, via restoring MMP function.

### TLB Suppressed H_2_O_2_-Induced Accumulation of Mitochondrial ${\text{O}}_{2}^{•-}$ and Improved Mitochondrial Enzyme Activities

**Figure 3:**
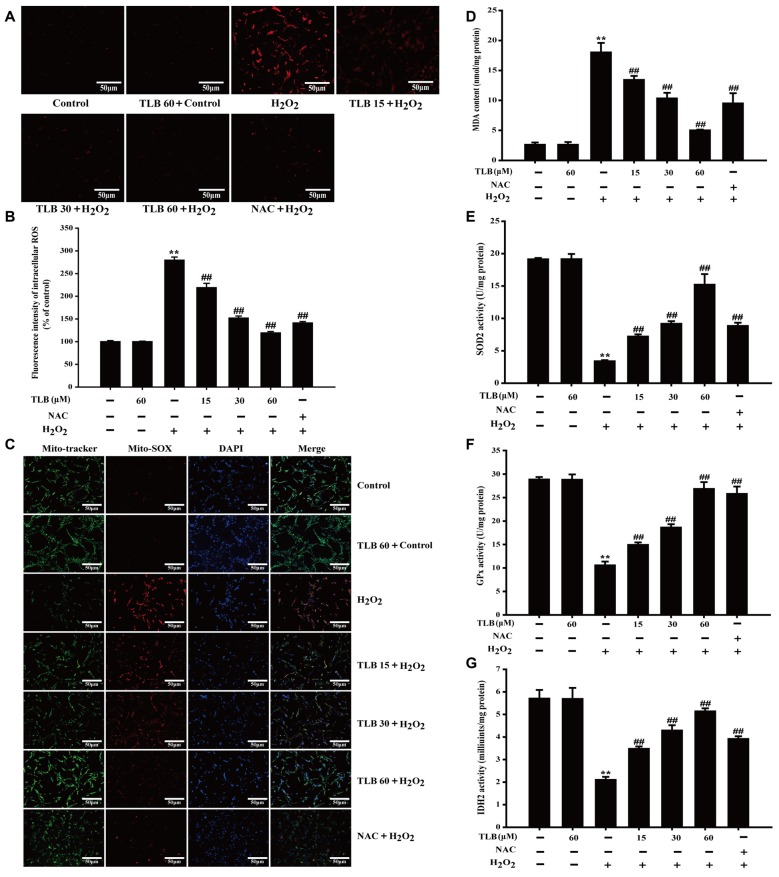
TLB suppressed H_2_O_2_-induced accumulation of intracellular reactive oxygen species (ROS) and mitochondrial superoxide (${\text{O}}_{2}^{•-}$) and improved mitochondrial enzyme activities. After pre-treatment of PC12 with or without TLB for 1 h, cells were treated with 400 μM H_2_O_2_ for another 48 h. **(A)** Intracellular ROS production was determined using 2’,7’-dichlorodihydrofluorescein diacetate (DCFH-DA) staining. Magnification, 200×; scale bar = 50 μm. **(B)** The mean fluorescence intensity of intracellular ROS was quantified using Image-Pro Plus software. **(C)** Mitochondrial ${\text{O}}_{2}^{•-}$ production was determined using MitoSOX Red staining. MDA content and enzymatic activities of superoxide dismutase 2 (SOD2), glutathione peroxidase (GPx), isocitrate dehydrogenase 2 (IDH2) were determined using according kits. Magnification, 200×; scale bar = 50 μm. **(D)** MDA content. **(E)** SOD2 activity. **(F)** GPx activity. **(G)** IDH2 activity. Data were presented as mean ± SD of three independent experiments. **P < 0.01 vs. untreated control cells; ^##^*P* < 0.01 vs. H_2_O_2_-treated cells.

Accumulating excessive ROS is considered to be one of the major causes of cell injury induced by H_2_O_2_. The level of intracellular ROS was determined using staining with DCFH-DA fluorescent probe in PC12 cells. The results showed that 400 μM H_2_O_2_ led to a dramatical augment in fluorescence intensity (279%) in the PC12 cells than that of control. However, the increase in ROS was dramatically inhibited by pre-treatment with TLB at different concentrations (219%, 152%, 119%, respectively; *F*_(6,14)_ = 523.086, *P* < 0.001; Figures 3A,B). Since mitochondria are the main source of ROS, determining the fate of cells through regulating multiple signal transduction pathways and mitochondria are also vulnerable targets. The protective effect of TLB on regulation of mitochondrial homeostasis was detected by MitoSOX Red staining, which is a specific fluorescent probe for detection of the mtROS generation, owing to MitoSOX Red is easily oxidized by ${\text{O}}_{2}^{•-}$ and displays red fluorescence within mitochondria. H_2_O_2_ markedly promoted mitochondrial ${\text{O}}_{2}^{•-}$ production than that of control. This effect was dramatically mitigated by TLB pre-treatment (Figure 3C). Furthermore, MDA, a marker of lipid peroxidation was measured. The data showed that H_2_O_2_ remarkably increased MDA content, while the increase in MDA was dramatically attenuated by TLB pre-treatment (*F*_(6,14)_ = 97.404, *P* < 0.001; Figure 3D). In addition, the results revealed that H_2_O_2_ significantly decreased enzymatic activities of SOD2 (*F*_(6,14)_ = 201.632, *P* < 0.001), GPx (*F*_(6,14)_ = 151.201, *P* < 0.001) and IDH2 (*F*_(6,14)_ = 72.049, *P* < 0.001), whereas TLB pre-treatment sharply increased these enzymatic activities (Figures 3E–G). These findings indicated that TLB protected the neuronal cells against oxidative stress through attenuating excessive intracellular ROS and mitochondrial ${\text{O}}_{2}^{•-}$ and its antioxidant role.

### TLB Ameliorated Mitochondrial Respiratory Chain Injury Induced by H_2_O_2_ Through Activated Sirt3

**Figure 4:**
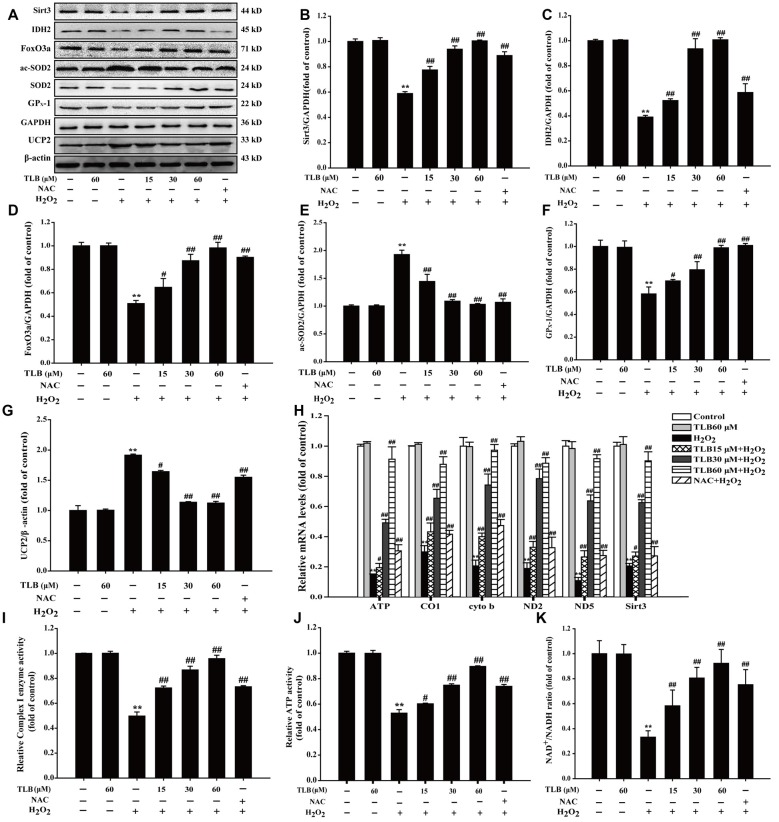
Mitochondrial respiratory chain and silent mating-type information regulation 2 homolog 3 (Sirt3) pathway mediated the protective effects of TLB in PC12 cells. Following pre-treatment with or without TLB for 1 h and incubated with 400 μM H_2_O_2_ for further 48 h, and the mitochondria in each group were isolated and purified. **(A)** Representative Western blots were shown for Sirt3, IDH2, forkheadboxO3a (FoxO3a)-, ac-SOD2, GP_X_-1 and uncoupling protein 2 (UCP2) proteins. **(B)** Quantitation of Sirt3 protein.** (C)** Quantitation of IDH2 protein. **(D)** Quantitation of Foxo3a protein. **(E)** Quantitation of ac-SOD2 protein. **(F)** Quantitation of GP_X_-1 protein. **(G)** Quantitation of UCP2 protein. **(H)** mRNA level of Sirt3 and mtDNA genes. **(I)** The activity of mitochondrial respiratory chain complex I. **(J)** The activity of adenosine triphosphate (ATP) ** (K)** Nicotinamide adenine dinucleotide (NAD)^+^/NADH ratio. Data were presented as mean ± SD of three independent experiments. **P < 0.01 vs. untreated control cells; ^#^*P* < 0.05, ^##^*P* < 0.01 vs. H_2_O_2_-treated cells.

The results showed that H_2_O_2_ down-regulated Sirt3, FoxO3a, IDH2 and GP_X_-1 expressions and up-regulated acetyl-SOD2 (ac-SOD2) and UCP2 expressions. However, pre-treatment with TLB was found to hinder the decreased expressions in Sirt3, FoxO3a, IDH2 and GP_X_-1 (*F*_(6,14)_ = 159.866, *P* < 0.001; *F*_(6,14)_ = 81.005, *P* < 0.001; *F*_(6,14)_ = 124.004, *P* < 0.001; *F*_(6,14)_ = 33.770, *P* < 0.001) and the increased in ac-SOD2 and UCP2 expressions (*F*_(6,14)_ = 90.023, *P* < 0.001; *F*_(6,14)_ = 821.299, *P* < 0.001; Figures 4A–G). Besides, TLB also upregulated both the mRNA level of Sirt3 (*F*_(6,14)_ = 198.419, *P* < 0.001) and mtDNA genes including ATP6, CO1, Cytb, ND2 and ND5 (*F*_(6,14)_ = 151.287, *P* < 0.001; *F*_(6,14)_ = 85.522, *P* < 0.001; *F*_(6,14)_ = 37.874, *P* < 0.001; *F*_(6,14)_ = 18.501, *P* < 0.001; *F*_(6,14)_ = 126.259, *P* < 0.001; Figure 4H). Furthermore, the mitochondrial ETC is considered to be the prime source of ROS and electrons can also react prematurely with oxygen at sites in the ETC to form ${\text{O}}_{2}^{•-}$/H_2_O_2_ (Murphy, 2009). The results showed that H_2_O_2_ not only decreased the enzyme activity of Complex I, which is often regarded as the major site of mitochondrial ${\text{O}}_{2}^{•-}$ production, but also reduced ATP activity and NAD^+^/NADH ratio. Whereas, pre-treatment with different concentrations of TLB efficiently improved Complex I activity, ATP activity and NAD^+^/NADH ratio, respectively (*F*_(6,14)_ = 23.265, *P* < 0.001; *F*_(6,14)_ = 234.113, *P* < 0.001; *F*_(6,14)_ = 129.227, *P* < 0.001, respectively; Figures 4I–K). These results indicated that TLB pre-treatment activated mtDNA transcription, thereby enriched Sirt3 and its downstream proteins, and then led to ameliorate mitochondrial ETC injury triggered by H_2_O_2_.

### TLB Activated AMPK Signaling Pathway in PC12 Cells

**Figure 5:**
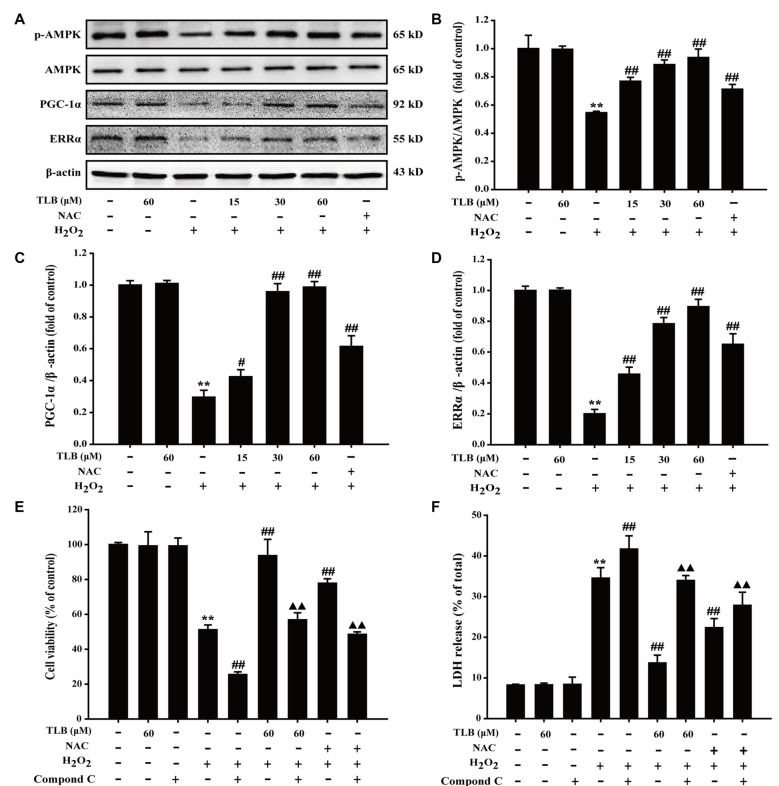
The role of adenosine monophosphate-activated protein kinase (AMPK) signaling pathway in the protective effect of TLB on H_2_O_2_-induced PC12 cells injury.** (A)** Representative Western blot were shown for AMPK, peroxisome proliferator-activated receptor gamma coactivator 1-alpha (PGC-1α) and ERRα proteins.** (B)** Quantitation of phosphorylation of AMPK protein. The relative optical density was normalized to AMPK. **(C)** Quantitation of PGC-1α protein.** (D)** Quantitation of ERRα protein. AMPK-specific inhibitor significantly abolished the protective effect of TLB. **(E)** Cell viability was determined using MTT assay.** (F)** LDH release was determined using an LDH release assay. Data were presented as mean ± SD of three independent experiments. **P < 0.01 vs. untreated control cells; ^#^*P* < 0.05, ^##^*P* < 0.01 vs. H_2_O_2_-treated cells; ^▲▲^*P* < 0.01 vs. TLB + H_2_O_2_-treated cells.

The results showed that H_2_O_2_ decreased p-AMPK level, PGC-1α and ERRα protein expressions, whereas those effects were reversed after pre-treated by TLB (*F*_(6,14)_ = 80.733, *P* < 0.001; *F*_(6,14)_ = 153.250, *P* < 0.001; *F*_(6,14)_ = 156.173, *P* < 0.001; Figures 5A–D), implicating that AMPK-PGC-1α-ERRα signaling pathway was activated by TLB during the neuronal cell injury challenged by oxidative stress. Moreover, the role of AMPK was further determined using Compound C, an AMPK inhibitor. The results showed that Compound C significantly abolished TLB-induced increase in cell viability and decreased TLB protection over H_2_O_2_ cytotoxicity, as evidenced by MTT and LDH assay, respectively (*F*_(8,18)_ = 99.300, *P* < 0.001; *F*_(8,18)_ = 143.943, *P* < 0.001; Figures 5E,F).

### TLB Activated Nrf2 Signaling Pathway in PC12 Cells

**Figure 6:**
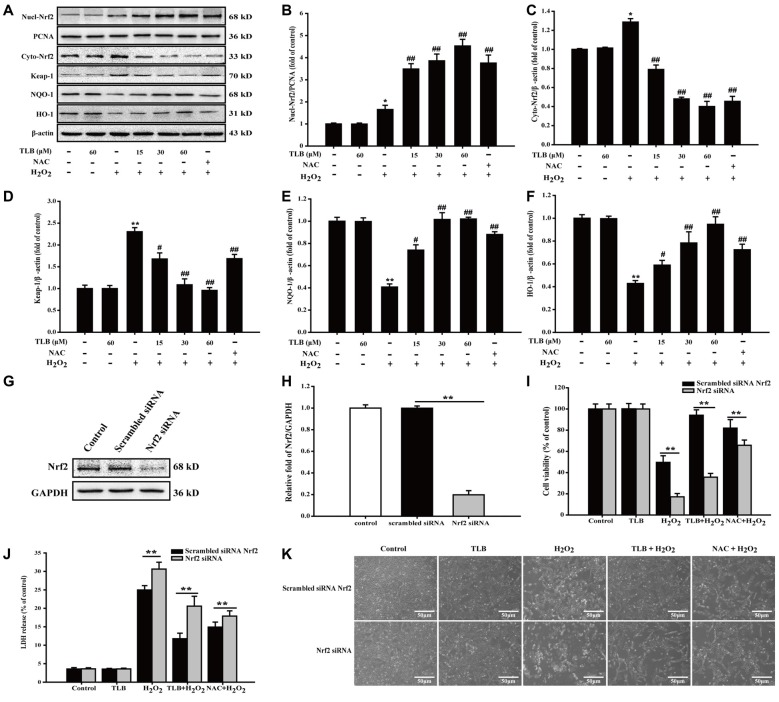
The role of nuclear factor erythroid 2-related factor 2 (Nrf2) signaling pathway in the protective effect of TLB on H_2_O_2_-induced PC12 cells injury.** (A)** Representative Western blot were shown for Nrf2, Keap-1, NQO-1 and HO-1 proteins. **(B)** Quantitation of nuclear Nrf2 protein. **(C)** Quantitation of cytoplasmic Nrf2 protein. **(D)** Quantitation of Keap-1 protein. **(E)** Quantitation of NQO-1 protein. **(F)** Quantitation of HO-1 protein. Nrf2 knockdown markedly abolished the cytoprotective effects of TLB in PC12 cells. **(G)** Representative Western blot was shown for Nrf2 siRNA. **(H)** Quantitation of Nrf2 siRNA protein.** (I)** Cell viability was determined in PC12 cells transfected with or without Nrf2 siRNA. **(J)** LDH release was determined in PC12 cells transfected with or without Nrf2 siRNA. **(K)** Morphological changes was observed in PC12 cells transfected with or without Nrf2 siRNA. Magnification, 200×; scale bar = 50 μm. Data were presented as mean ± SD of three independent experiments. *P < 0.05, **P < 0.01 vs. untreated control cells; ^#^*P* < 0.05, ^##^*P* < 0.01 vs. H_2_O_2_-treated cells.

To further explore the effects of TLB on Nrf2 and its downstream genes, PC12 cells were pre-treated with indicated concentrations of TLB for 48 h. The results showed that TLB increased Nrf2 expression in the nucleus, compared with H_2_O_2_ exposure alone (*F*_(6,14)_ = 42.307, *P* < 0.001). In contrast, Nrf2 expression decreased in the cytoplasm (*F*_(6,14)_ = 254.231, *P* < 0.001), suggesting that TLB promoted Nrf2 nuclear translocation. To further investigate the effects of TLB on Nrf2 downstream target genes, Keap-1, NQO-1 and HO-1 protein expressions were determined. The results showed that TLB decreased the Keap-1 expression and increased NQO-1 and HO-1 expressions, compared with H_2_O_2_ exposure alone (*F*_(6,14)_ = 11.292, *P* < 0.001; *F*_(6,14)_ = 58.629, *P* < 0.001; *F*_(6,14)_ = 14.611, *P* < 0.001; Figures 6A–F). In addition, SiRNA was used to further confirm that TLB activated Nrf2 to protect PC12 cells against H_2_O_2_-induced injury. Compared with non-transfected control cells and scrambled siRNA transfected, Nrf2 siRNA treated group displayed lower levels of Nrf2 (*F*_(2,6)_ = 806.527, *P* < 0.001; Figures 6G,H). Without any oxidative stress stimulus, inhibition of Nrf2 exhibited no effect on cell viability and cytotoxicity. However, under conditions of being challenged by H_2_O_2_, the cell viability and cytotoxicity of Nrf2 siRNA transfected cells were markedly lowered and elevated, respectively (*F*_(4,10)_ = 370.761, *P* < 0.001; *F*_(4,10)_ = 494.406, *P* < 0.001), compared with that of scrambled siRNA transfected cells. Whereas, the protective effect of TLB was dramatically blocked by the Nrf2 siRNA (*F*_(4,10)_ = 63.074, *P* < 0.001; *F*_(4,10)_ = 574.052, *P* < 0.001; Figures 6I–K).

### AMPK/Nrf2/Sirt3 Axis Was Involved in the Neuroprotective Effects of TLB on H_2_O_2_-Induced PC12 Cells Injury

**Figure 7:**
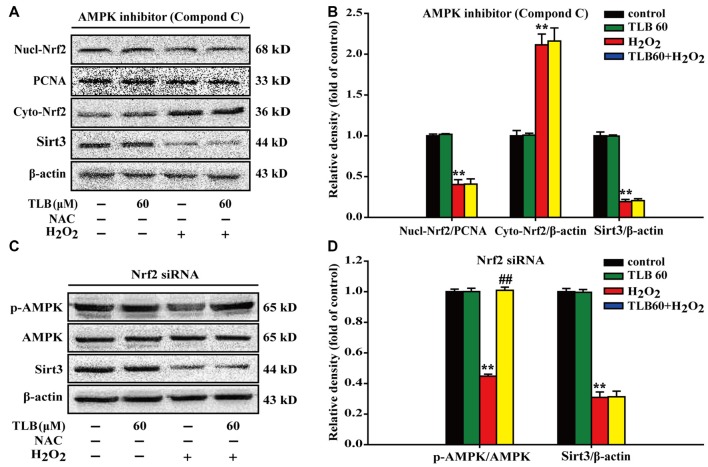
The role of AMPK/Nrf2/Sirt3 axis in the protective effect of TLB on H_2_O_2_-induced PC12 cells injury. AMPK inhibitor was added to the cell cultures 1 h or Nrf2 was knocked down by Nrf2 siRNA transfection as mentioned in the “Materials and Methods” section. Following pre-treatment with or without TLB for 1 h and incubated with 400 μM H_2_O_2_ for further 48 h. **(A)** Representative Western blot were shown for Nucl-Nrf2, Cyto-Nrf2 and Sirt3 proteins.** (B)** Quantitation of Nucl-Nrf2, Cyto-Nrf2 and Sirt3 proteins. **(C)** Representative Western blots were shown for p-AMPK and Sirt3 proteins.** (D)** Quantitation of p-AMPK/AMPK and Sirt3. Data were presented as mean ± SD of three independent experiments. **P < 0.01 vs. untreated control cells; ^##^*P* < 0.01 vs. H_2_O_2_-treated cells.

To further investigate the relationship of AMPK, Nrf2 and Sirt3, Nrf2 siRNA or p-AMPK inhibitor were applied. The results showed that p-AMPK inhibitor abrogated the protective effects of TLB on H_2_O_2_-induced changes of Nucl-Nrf2 (*F*_(3,8)_ = 193.076, *P* < 0.001), Cyto-Nrf2 (*F*_(3,8)_ = 117.987, *P* < 0.001) and Sirt3 (*F*_(3,8)_ = 805.150, *P* < 0.001; Figures 7A,B). Moreover, knockdown of Nrf2 almost deterred the protective effects of TLB on H_2_O_2_-induced change of Sirt3 (*F*_(3,8)_ = 321.826, *P* < 0.001), but did not affect p-AMPK level (*F*_(3,8)_ = 831.253, *P* < 0.001) in PC12 cells (Figures 7C,D). These findings indicated that AMPK/Nrf2/Sirt3 axis plays an essential role for the effect of TLB on regulation of mtROS homeostasis in PC12 cells.

## Discussion

**Figure 8:**
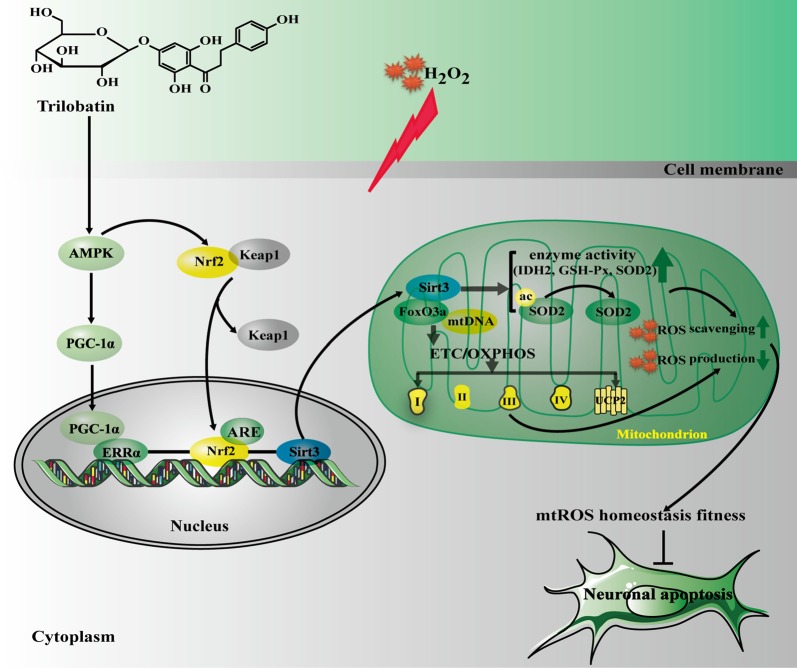
The schematic presentation elucidating proposed mechanisms that TLB protects PC12 cells against oxidative stress-induced injury. Excessivemitochondrial reactive oxygen species (mtROS) production is triggered by H_2_O_2_ leading to oxidative injury in neuronal cell, and even apoptosis. TLB mitigates overproduction of mtROS and amelioriates the damage of mitochondrial respiratory chain induced by H_2_O_2_ through mediating the AMPK/Nrf2/Sirt3 axis.

The current study, for the first time, revealed that: (1) TLB, a novel small molecule monomer, derived from *Lithocarpus polystachyus* Rehd exerted neuroprotective effects against oxidative stress-induced neuronal cell apoptosis; (2) the beneficial effects of TLB was owed to attenuation of mitochondrial ETC injury and inhibition of ${\text{O}}_{2}^{•-}$ generation within the mitochondria; (3) TLB not only activated AMPK/Sirt3 signaling pathway, but also facilitated nuclear translocation of transcription factor Nrf2 and in turn promoted antioxidant gene expression; and (4) Most importantly, the findings of current study, revealed that Nrf2 is the downstream of AMPK and upstream of Sirt3, respectively. AMPK/Nrf2/Sirt3 axis plays an imperative role in the neuroprotective effect of TLB on the oxidative injury in neuronal cells (Figure 8).

Oxidative stress is considered as a valid therapeutic strategy for neurological diseases since the particular vulnerability of the brain to oxidative injury and high levels of ROS may be involved in neuropathology. Oxidative stress occurs under the condition of imbalance of cellular redox homeostasis, and it is further related to a lot of pernicious outcome for the neuronal cells such as lipid peroxidation or even apoptosis. In the present study, H_2_O_2_, the most stable ROS, was used to induce oxidative stress in PC12 cells due to its characteristic with an extracellular messenger and an intercellular one, which alter several proteins, thus leading to mitochondrial injury and even cell apoptosis. Here, the results demonstrated that H_2_O_2_ triggered cell injury, accompanied by mitochondrial ${\text{O}}_{2}^{•-}$ generation and cell apoptosis via activating caspases and releasing proapoptotic factors including Bax and Cyto *c* from mitochondria, consisting with our previous study (Gao et al., 2017). Whereas, these effects were reversed by TLB pre-treatment. These findings highlighted that TLB-regulated pharmacological preconditioning effect in neuronal cells, at least in partly, through Caspase 3-dependent apoptosis pathway.

Excessive mtROS production can lead to oxidative damage to cellular ingredients such as lipids, proteins and mitochondrial DNA and induce cellular senescence. Reduced ETC/OXPHOS ability can lead to cellular energy deprivation such as reduced ATP charge, altered mtROS production, or loss of MMP, with the precise consequence dictating the specific mitochondrial stress-signaling response (Song et al., 2017). Thus, it appears possible that the beneficial effect of TLB might be related to regulation of mtROS homeostasis. To this end, mitochondrial ${\text{O}}_{2}^{•-}$ level and ETC were determined in the present study. As expected, the results suggested that TLB attenuated mitochondrial ${\text{O}}_{2}^{•-}$ generation, thereby restored MMP as evidenced by MitoSOX Red dying and rhodamine 123 staining. Furthermore, evidence indicates that Complex I, the major source of spontaneous ${\text{O}}_{2}^{•-}$, is the point of entrance anion production in the mitochondrial ETC for NADH reducing equivalents, and it not only engenders ROS but also maintains the proton gradient across the mitochondrial inner membrane and is needed for ATP synthesis. Moreover, since brain neurons are sensitive to deficiencies in OXPHOS, and complex I is particularly larger than other cell types (Pinto et al., 2012). Thus, defects in complex I are linked with multiple human neurological diseases (Papa and De Rasmo, 2013). Our results demonstrate that oxidative stress induced excessive ROS production by disrupting complex I, thereby reducing ATP synthesis and NAD^+^/NADH ratio, which is in consistent with the theory that complex I is the first step in energy metabolism and redox pathways (Martinvalet et al., 2008). However, TLB reversed these effects, further confirmed that TLB protected against oxidative stress-induced neuronal cell injury through inhibition of mitochondrial dysfunction. Additionally, the deleterious effects of oxidative stress can be deterred by antioxidant enzymes. Our findings manifested that TLB also enhanced the mitochondrial antioxidant enzyme activities of SOD2, GPx-1 and IDH2 to eliminate overproduction of ROS. Intriguingly, SOD2 is not only the forefront antioxidant enzyme that protects cells against excessive ROS generated in the mitochondria, but also a direct substrate of Sirt3, which is a NAD^+^-dependent deacetylase localized in the mitochondrial matrix, and can mediate ROS levels also by deacetylating ETC complexes to lower mtROS production and maximize ATP synthase (Karnewar et al., 2018). As expected, TLB activated Sirt3, thereby facilitated deacetylation of SOD2 in mitochondria and triggered its downstream gene IDH2 to scavenge mitochondrial ${\text{O}}_{2}^{•-}$. Furthermore, a body of evidence suggests that Sirt3 binds mtDNA through regulating FoxO3a, thereby elevated ETC capacity and ATP synthesis. Consistent with that theory, our findings revealed that TLB not only enriched Sirt3 in mitochondria, but also triggered mtDNA transcription, and then regulated redox status in neuronal cells. Moreover, it is noteworthy that UCP2, a new member of the mitochondrial uncoupling protein family, which negatively controls MMP and ATP production to attenuate mtROS when neuronal cells were challenged by oxidative stress (Suzuki et al., 2015). In keeping with the theory, the results in this study revealed that H_2_O_2_ increased UCP2 expression, while, TLB blocked the increase in UCP2 expression, further confirmed TLB maybe targeted mitochondria to mediate proper balance between the productions of mitochondrial energy and mtROS. However, the underlying molecular mechanisms need to be further investigated.

To elucidate the mechanism of TLB-triggered Sirt3 activation, we investigated the activation of AMPK, PGC-1α, and ERRα. AMPK, a ubiquitous serine/threonine protein kinase, is known to regulate cellular metabolic balance, and it is activated in response to disruptions in cellular energy such as decreases in cellular ATP. Recently, AMPK has been reported to influence mtROS homeostasis by initiating PGC-1α, a downstream effector of AMPK. Hence, AMPK plays a crucial role in intracellular metabolism and may be a potential therapeutic target for mtROS-related neurological diseases. Furthermore, previous evidence demonstrates that PGC-1α acts as an endogenous regulator of Sirt3 and activates Sirt3 by triggering ERRα to cause ERRα coupling with ERRE in the Sirt3 promoter region (Zhou et al., 2014). We therefore made an assumption that TLB controls mtROS homeostasis via activating the AMPK-PGC-1α- Sirt3 axis. As expected, the results indicated that H_2_O_2_ decreased p-AMPK, in keeping with the theory that oxidative stress acts as a trigger for AMPK (Wu et al., 2018). Whereas, TLB promoted AMPK phosphorylation, thereby triggered activation of PGC-1α and ERRα, suggesting that AMPK-PGC-1α-Sirt3 axis was necessary for the protective effect of TLB on regulation of mtROS homeostasis in neuronal cells. Intriguingly, interaction between AMPK and Nrf2 also appears to play a positive role of oxidative stress-induced neuron demise, and Nrf2 interacts with PGC-1α to mediate crucial signaling pathway within mitochondria and triggers the activation of Sirt3 to exert neuroprotective effect under oxidative stress condition (Shah et al., 2016; Wang et al., 2017). Nrf2 is known as a transcription factor and its inactive form is kept in the cytoplasm bound to Keap-1. When the cell is in an oxidative stress condition, active sites of cysteine residues of Keap-1 were oxidized, thereby prevented Keap-1 from bonding with Nrf2. Then, Nrf2 translocates into the nucleus and bonds to ARE including HO-1 and NQO-1, which are the crucial genes in the brain and is activated by Nrf2 to protect against neurons cell injury (Shetty et al., 2017). We thus focused on the role of Nrf2 in-depth. Our findings demonstrated that TLB decreased Keap-1, which is considered as cytoplasmic inhibitor of Nr2, and then promoted Nrf2 translocation from cytoplasm into nucleus, and improved its antioxidant genes. Most importantly, knockdown of Nrf2 partially abolished the protection of TLB, further confirmed that the protective mechanism of TLB is, at least in partly, through activating Nrf2. Based on the above findings, it is reasonable assumed that AMPK/Nrf2/Sirt3 axis was involved in the salutary effects of TLB on H_2_O_2_-induced PC12 cell injury. Intriguingly, we uncovered that Nrf2 and Sirt3 were the downstream genes of AMPK, and Nrf2 was the upstream of Sirt3, evidenced by AMPK inhibitor or knockdown of Nrf2. Importantly, as expected, TLB exerted neuroprotective effects on H_2_O_2_-induced neuronal cell injury, at least in partly, through mediating AMPK/Nrf2/Sirt3 axis. Together, these findings suggested that there exists crosstalk between oxidative stress, mitochondria, and apoptosis, however, the precise underlying mechanisms of reciprocity between AMPK, Nrf2 and Sirt3 were needed to further study.

Additionally, since TLB is a novel compound, we did primary research to confirm its antioxidative effects in PC12 cells, which was accepted as a common model for studying of neuronal properties (Liu et al., 2018). Although we have confirmed the neuroprotective effects of TLB in PC12 cells after oxidative stress insults, further studies are necessary to determine the neuroprotective role of TLB in other types of cells such as human neuron-like cells SH-SY5Y or mouse/rat primary neurons. Moreover, considering TLB is a small molecule monomer, we hypothesize that it could overcome blood brain barrier (BBB) in *vivo* although there is no direct evidence. Thus, whether TLB could cross the BBB and confer neurological diseases such as ischemic cerebral stroke *in vivo* is imperative to explore in our is further studies.

## Conclusion

In summary, the results of this study demonstrate that TLB exerts its salutary effects on oxidative stress-induced neuronal apoptosis via mediating mtROS homeostasis and the AMPK/Nrf2/Sirt3 signaling pathway. To our knowledge, this is the first indication that TLB exhibits antioxidant properties to eliminate overproduction of mtROS and plays an imperative role in oxidative stress-induced neuronal demise, suggesting that TLB may be a promising natural radical scavenger for the prophylaxis and treatment of neurological diseases.

## Author Contributions

QG and JS designed the experimental approaches. JG performed all the other studies described herein, except the Western blot experiments conducted by SL and FX, Annexin V-FITC/PI staining by YL, and Quantitative real-time PCR analysis conducted by CL and YD. JG wrote the manuscript with the help from QG.

## Conflict of Interest Statement

The authors declare that the research was conducted in the absence of any commercial or financial relationships that could be construed as a potential conflict of interest.
